# Exploration of Psychological Mechanism of Smartphone Addiction Among International Students of China by Selecting the Framework of the I-PACE Model

**DOI:** 10.3389/fpsyg.2021.758610

**Published:** 2021-11-12

**Authors:** Anam Mehmood, Tianyi Bu, Erying Zhao, Viktoriia Zelenina, Nikishov Alexander, Wantong Wang, Sultan Mehmood Siddiqi, Xiaohui Qiu, Xiuxian Yang, Zhengxue Qiao, Jiawei Zhou, Yanjie Yang

**Affiliations:** ^1^Department of Medical Psychology, Psychology and Health Management Center, Harbin Medical University, Harbin, China; ^2^Department of Nutrition and Food Hygiene, College of Public Health, Harbin Medical University, Harbin, China

**Keywords:** I-PACE model, international student, smartphone addiction, lonelineness, stress

## Abstract

The I-PACE (interaction of person-affect-cognition-execution) model explains that the causes of addiction are the result of individual susceptibility (genetic and personality), psychopathological factors (negative emotions), and cognitive and affective factor interaction. The issue of smartphone addiction and its emerging effects are now becoming an essential social enigma. This study is aimed at exploring how personal, affective, cognitive, and execution factors accelerate the mechanism of smartphone addiction among international students. Randomly selected, six hundred international students have constituted the population for our study. All participants were asked to complete self-administered questionnaires. The questionnaire included demographics (gender, place of stay, educational level, and reason for smartphone usage), Mobile Phone Addiction Index, Loneliness Scale (UCLA), Rosenberg Self-Esteem Scale, Beck Depression Inventory, Perceived Stress Scale, Eysenck Personality Questionnaire, and Simplified Coping Style Questionnaire. Statistical analysis was performed using SPSS. 20.3% (*n* = 122) of international students are agonized with smartphone addiction, while 79.7% (*n* = 478) use smartphones at an average level. Students’ place of stay, neuroticism personality, social desirability, self-esteem, loneliness, depression, perceived stress, and passive coping are associated with smartphone addiction. Loneliness and depression show a strong positive significant correlation, among other variables while loneliness, neurotic personality, depression, low self-esteem, stress, and passive coping are risk factors for smartphone addiction. This study reveals that international students are a high-risk group for smartphone addiction. It has a great deal of impact on students’ behavior and psyche. Multiple social, psychological, affective, and cognitive factors affect smartphone addiction. It would be beneficial to direct the students to limit their phone usage and indulge in other healthy physical activities to complete academic goals.

## Introduction

A smartphone is the groundbreaking technology of our time. A smartphone is a portable device with ever-increasing advanced features, including a portable computer system, recording and relaying devices, and enhanced memory. These characteristics make the use and ownership of smartphones a common social phenomenon (Smartphone, 2016). According to an international analysis report (2011), smartphones’ penetration rate among adults aged 21–30 in China was 68.4%, increasing with each passing year (Xinhua Net, 2011). As per the New York Times (2017), adults and teenagers check their smartphones 150 times a day, or after every 6 min (Kiran et al., 2019). The latest Filipino survey found that the prevalence of smartphone addiction was as high as 60% in the adult population (Buctot et al., 2020). There is no doubt that smartphones bring convenience and positive results. On the other hand, it has been observed that, especially among the young population, its excessive use is causing addiction at an alarming rate. The issue of smartphone addiction and its’ emerging effects are now becoming an essential social conundrum.

Furthermore, Smartphone addiction, a kind of behavioral addiction, refers to the uncontrollable overuse of smartphones, and preoccupation with it leads to obsession. It may be due to tackling problems in real life, concealment of the actual use from friends and family, or abating the withdrawal symptoms. The addiction prompts continuing usage even after knowing adverse corollaries. Smartphone addiction is also distinguished as a disorder of smartphone usage or smartphone dependence (Billieux, 2012; Billieux et al., 2015).

College students experience high levels of stress which may be due to the frequency of exams, long and tedious academic coursework, and concern about the future (Gazzaz et al., 2018). When confronting with these stresses, students are more likely to turn to their smartphones for stress relief. College students also use more smartphones because of extra leisure time and less parental control. Due to smartphones’ frequent use, Students might become addicted to the virtual world and disengaged from real life, affecting their physical and mental health (Schwebel et al., 2012). A Chinese survey (2016) has found that 21.3% of college students have smartphone addiction (Long et al., 2016). In addition, a survey report from South Korea (2013) reported that 25.5% of college students were at risk of smartphone dependence (Jun, 2015). Therefore, smartphone addiction among students had already aroused widespread attention.

Regarding international students, Smartphones are invaluable for connecting with family and friends. International students become more vulnerable to smartphone addiction due to loneliness, cultural adaptation, and adjustment stress (Yeh and Inose, 2003; Lee and Rice, 2007; Poyrazli and Lopez, 2007; Wang, 2009; Poyrazli, 2014). As international students spend their time in a new culture far away from their families and hometown. They experience boredom, loneliness, stress, and anxiety about their family’s health. As a way of coping, they often use smartphones. Smartphone usage can alleviate their stress and worries, as well as academic burden, admiration, breakup, and so on (Lin et al., 2015).

However, smartphone addiction is likely to create a series of health problems such as distorted vision, neck stiffness, wrist or back pain (Kim and Kang, 2013; Lee et al., 2015) psychological troubles (Demirci et al., 2015) sleep disorders, interpersonal conflicts, academic failure, and other issues (Lam et al., 2009; Mok et al., 2014). Kim et al. (2015) investigated that smartphone addicts are most likely to have health-related issues, such as physical complaints (headache, dry eyes, and carpal tunnel syndrome) and psychological problems (fear, depression, sadness, and anxiety) (Küçer, 2008; Lee and Seo, 2014). A range of studies conducted on personality factors affecting smartphone overuse had concluded that self-esteem acts as a mediator (Wang et al., 2017) while neuroticism and extraversion personalities, and self-control play an intermediary role in Smartphone addiction (Cho et al., 2017). Also, some exploratory surveys have shown that depression and loneliness are factors leading to smartphone addiction (Mok et al., 2014; Demirci et al., 2015). Similarly, HyunChul Youn and his co-workers have confirmed that levels of depression are positively correlated with smartphone addiction (Choi et al., 2015). Stress is the risk factor of addictive behavior and cause of addiction recurrence (Sinha, 2008; Lam et al., 2009). Numerous explorations have discovered that there is a positive correlation between using smartphones and stress (Harwood et al., 2014), while various epidemiological researches have also reconnoitered the impact of coping styles on smartphone addiction. Cheng et al. pointed out that the lack of positive coping strategies led to smartphones’ excessive use (Cheng et al., 2015), and Tang et al. (2014) alleged that a negative coping style was significantly associated with the risk of smartphone addiction.

The formation and maintenance process of psychological mechanisms of smartphone addiction is still unclear. In 2014, Brand proposed the I-PACE (interaction of person-affect-cognition-execution) model; he believed that the causes of addiction were the result of individual susceptibility (genetic and personality), psychopathological factors (negative emotions), cognitive and affective factors interaction (Brand et al., 2014). This model was latterly used to study gambling addiction and Internet addiction (Dong et al., 2019; Ioannidis et al., 2019). I-PACE model is adopted because this theoretical model has the goal to summarize the main processes underlying both the development and the maintenance of smartphone addiction (Brand et al., 2016). This model also provides a theoretical basis for addictive behaviors and clinical practice (Brand, 2017).

The significance of our study let off from the fact that the number of smartphone users is seriously escalating which means that more and more individuals are expected to become smartphone addicts. Therefore, We attempt to hit this growing field of interest and expect to provide researchers with a theoretical framework on smartphone addiction. Another expected contribution of our study is the detection of the frequency of smartphone addiction among international students. This may provide awareness for rational use of the smartphone for both physical and mental health. We also hope to illuminate psychological therapy and counseling by edifying the characteristics of smartphone addicts and the relationship between smartphone addiction and several variables.

Based on the I-PACE model, the study aims to analyze the problems of smartphone addiction from multiple aspects. I-PACE model is elected to explain the personal (personality and self-esteem), affective (loneliness and depression), cognitive (stress perception), and execution (coping style) factors interaction in smartphone addiction. International students are selected as China is the world’s leading country and offers a research-oriented forum for international students worldwide (Ye, 2006; Zhang and Zhou, 2010; Henze and Zhu, 2012) and to learn about various cultures, psychosocial development, and multicultural competence. And there are only a few pieces of research on this ever-expanding population of international students. The research outcome will elucidate the psychological mechanism of the development and maintenance of smartphone addiction among international students in China. Moreover, will also provide a theoretical basis for the prevention and intervention of smartphone addiction among college students. This is the first study to explore the psychological mechanism of smartphone addiction based on the I-PACE model and targeted international students of Harbin, China The purpose of the present research is to explore the current status of smartphone addiction among college students based on different demographic characteristics, investigate the relationship between various components of I-PACE model and smartphone addiction, examine the effect of personality, biological psychology, psychopathology, and social cognition on smartphone addiction and to identify the risk factors of smartphone addiction.

## Materials and Methods

### Participants

This observational population study adopted cluster sampling to recruit international students with bachelor’s, master and doctoral programs living in Harbin, China. The participants who met the conditions for inclusion in this study were international students living only in Harbin, China, and participated voluntarily. Exclusion criteria include living outside Harbin, under the age of 18, voluntary problems, and jobholders. The study included 700 students, of which 100 chose to withdraw due to the exclusion criteria, so 600 students finally constituted the study sample. The average age of the participants was 23.63 years (SD = 3.862), with a range of 18–38 years. A total of 59.5% (*n* = 357) of the study population were male and 40.5% (*n* = 243) were female.

### Procedure

The data collection period was from August 2019 to December 2019. The well-trained graduate students sent complete questionnaires and an instruction package to different universities in Harbin, China. It was requested to give 25 to 30 min after class to complete the questionnaire package. Before participation, informed consent was distributed among all participants. Participants were informed that they were not obliged to participate; all responses were anonymous and they were free to refuse to answer any questions. Questionnaires were collected immediately after completion and checked to ensure quality and avoid errors. All records were kept in a locked computer and under strict supervision.

#### Informed Consent Statement

Informed consent was obtained from all subjects involved in the study.

#### Ethical Approval

Before data collection, ethical approval was obtained from the Institutional Review Board of the School of Public Health, Harbin Medical University. Before collecting data, participants were informed about the research and their role in participation.

#### Psychological Measures

A questionnaire package designed to obtain sociodemographic information (gender, place of stay, educational level, and reason for smartphone usage) was given to the participants. Furthermore, the participants were requested to complete the Mobile Phone Addiction Index, Loneliness Scale (UCLA), Rosenberg Self-Esteem Scale, Beck Depression Inventory, Perceived Stress Scale, Eysenck Personality, and Simplified Coping Style questionnaires. All questionnaires were in the self-report format.

#### Mobile Phone Addiction

Louis Leung of Hong Kong University developed Mobile Phone Addiction Index (Leung, 2008). This scale consists of 17 items that measure four dimensions of smartphone addiction: inability to control cravings (seven items), anxiety and feeling lost (four items), withdrawal and escape (three items), and productivity loss (three items). Participants answered these items on a 5-point scale (ranging from 1 = never to 5 = always). MPAI has good validity and reliability and is widely used in the diagnosis of smartphone addiction. The higher the score, the higher was smartphone addiction. In our research, the measure showed good reliability (Cronbach’s α = 0.86).

#### Personality

Personality was assessed using the Eysenck Personality Questionnaire (EPQ; Eysenck and Eysenck, 1975). The original English version contains 90 items; however, the Chinese version has 88 items because some unnecessary elements have been eliminated. The EPQ questions elicit dichotomous responses (e.g., “Do you have a lot of different hobbies?”). Expert translators translated the Chinese version of EPQ into the English language and used it to collect international data. EPQ measured individuals’ personalities across four dimensions: neuroticism/anxiety, psychoticism, extroversion, and social desirability (a lie scale). The measure presented good reliability in our study (Cronbach’s α is 0.89, 0.79, 0.81, and 0.82 for neuroticism/anxiety, psychoticism, extroversion, and social desirability, respectively).

#### Self-Esteem

The Rosenberg Self-Esteem Scale evaluated the respondent’s judgment of his or her self-worth (e.g., “On the whole, I am satisfied with myself”) (Rosenberg, 2015). This scale consists of 10 items. The participants rated each item on a 4-point scale ranging from strongly disagree to strongly agree, with higher scores indicating a higher level of self-esteem. In the current study, the measure showed good reliability (Cronbach’s α = 0.96).

#### Loneliness

A short form of the UCLA Loneliness Scale developed by Russell (1996) was used to access loneliness Respondents were asked to express how they feel about eight statements using a 4-point Likert-type scale, ranging from 0 = never to 3 = always (e.g., “How often do you feel unhappy doing so many things alone?”). The greater the score, the higher would be the loneliness. The measure exhibited good reliability in our survey (Cronbach’s α = 0.81).

#### Depression

The Beck Depression Inventory consists of 21 items to assess the severity of depression (Beck et al., 1961). Each question uses a 4-point scale to ask about the specific symptoms of the recent feelings (e.g., “sadness, hopelessness”). The total score indicated the degree of depression. In the current study, the measure showed good reliability (Cronbach’s α = 0.86).

#### Stress Perception

The Perceived Stress Scale consists of 14 items to evaluate stress perception (e.g., “Feeling nervous and stress?”) (Cohen et al., 1983). The participants rated each item on a 5-point Likert scale ranging from 1 = never to 5 = very much, with higher scores indicating a higher level of perceived stress. In the current study, the measure showed good reliability (Cronbach’s α = 0.94).

#### Coping Strategies

Coping style was measured by Simplified Coping Style Questionnaire (SCSQ; Xiao and Xu, 1996). It consists of 20 items, and respondents responded on a four-point Likert scale rated from 0 (never) to 3 (always) (e.g., “Let the pressure off through work or study and activities”). It measured both active coping and passive coping styles. Positive coping style consisted of prosocial approach behaviors that focused on the stressor itself which included 12 items. The negative coping style consisted of some avoidant behaviors that were not focused on the negative events which included eight items. For the current study, the measures showed good reliability (Cronbach’s α is 0.88 and 0.84 for positive and negative coping styles, respectively).

### Statistical Analysis

Statistical analysis was performed using SPSS 22.0. Both smartphone addicts and non-addicts were included in our study. Demographic data including age, gender, place of stay, educational level, and smartphone usage reasons were recorded as numbers and percentages. The proportion of missing data was less than 1%, which was handled through mean imputation. A chi-square test was used to compare smartphone addiction in different demographic variables, and correlation analysis was used to measure the strength of the relationship between variables (Prematunga, 2012; McHugh, 2013). Bivariate logistic regression was used to analyze the risk factors of smartphone addiction (Denis, 2018).

## Results

### Sociodemographic Data of Participants

The present study comprised 600 international students from Harbin, China. The incidence of smartphone addiction was 59 and 41% in male and female students, respectively. Out of these participants, 6.3% lived with parents/family, 83.7% alone in the dormitory, and 10% alone in a rented apartment. 61.7% (*n* = 370) of the participants were bachelor students, 20.3% (*n* = 122) were studying master courses and 18% (*n* = 108) were Ph.D. students. 26.0% of the subjects used smartphone for social media, 18.2% for calls, 49.3% for internet, 0.3% for mail, 0.2% for short message services, 0.8% for playing games, 0.7% for making videos/pictures and 4.5% used smartphone for other purposes (Table 1).

**TABLE 1:** Smartphone addiction based on demographics.

Demographic variables	Smartphone addicts *n* (%)	Smartphone non-addicts *n* (%)	χ2	*P*-value
**Gender**				
Female	50 (41%)	193 (40.3%)	0.204	0.903
Male	72 (59%)	285 (59.7%)		
**Place of stay**				
With parents/family	11 (9%)	27 (5.6%)	13.151	0.011
Alone in dormitory	100 (82%)	402 (84.2%)		
Alone in apartment	11 (9%)	49 (10.2%)		
**Educational level**				
Bachelor	82 (67.2%)	288 (60.3%)	3.712	0.446
Masters	22 (18%)	100 (20.9%)		
Ph.D.	18 (14.8%)	90 (18.8%)		

### Prevalence of Mobile Phone Addiction

20.3% (*n* = 122) of international students was smartphone addicts while 79.7% (*n* = 478) used smartphone at normal level.

### Comparison Between Demographic Variables and Smartphone Addiction

Table 1 explains the comparison between demographic variables and smartphone addiction. Students’ place of stay (χ2 = 13.51, *p* = 0.036) had a significant positive impact on smartphone addiction while gender and level of education had no significant correlation for smartphone addiction.

### Strength of the Relationship Between Smart Phone Addiction and Components of I-PACE Model

Personality components (extrovert personality, psychoticism personality, lying personality, and self-esteem) were negatively correlated with smartphone addiction, while active coping revealed no significant correlation for smartphone addiction. In contrast, neuroticism personality, affective (loneliness and depression), cognitive (perceived stress), and executive (passive coping style) components of the I-PACE model had a significant positive correlation with smartphone addiction. Loneliness and depression presented a strong positive relationship with smartphone addiction, among other variables (Table 2).

**TABLE 2:** Pearson correlation matrix for observed variables.

Variables	1	2	3	4	5	6	7	8	9	10	11
1. Smartphone addiction	–										
2. Extrovert personality	−0.018**	–									
3. Neuroticism personality	0.214**	0.385**	_								
4.psychotics personality	−0.017**	0.236**	0.339**	–							
5. Lying personality	−0.013**	−0.073**	−0.185**	–0.057	–						
6. Self-esteem	−0.151**	0.003**	–0.011	0.089**	0.081**	–					
7. Loneliness	0.327**	−0.10**	−0.166**	−0.225**	–0.021	−0.081**	–				
8.Depression	0.368**	−0.12**	−0.143**	−0.135**	–0.005	–0.213	0.382**	–			
9. Perceived stress	0.113**	−0.115**	−0.233**	−0.257**	0.042	–0.039	0.323**	0.297**	–		
10. Active coping	–0.063	–0.052	−0.106**	−0.061**	0.004	−0.067**	0.140**	0.169**	–0.006	–	
11. Passive coping	0.107**	0.025	–0.009	0.037	0.02	−0.095**	–0.071	0.008	−0.088**	0.036	–

**Correlation is significant at the 0.05 level.

### Predicting Smartphone Addiction

Based on a bivariate logistic model, the possibility of smartphone addiction was significantly higher in those international students who have loneliness, neurotic personality, stress, depression, low self-esteem, and passive coping style (Table 3).

**TABLE 3:** Risk factors for development and maintenance of smartphone addiction.

Variables	*B*	SE	*P*-value	Exp (*B*)	LC	UC
Intercept	0.960	1.223	0.433			
Extrovert personality	0.051	0.047	0.275	1.053	0.960	1.154
Neuroticism personality	0.049	0.040	0.031	1.050	0.971	1.136
Psychoticism personality	–0.068	0.053	0.198	1.071	0.965	1.188
Lying personality	–0.072	0.051	0.052	1.074	0.972	1.187
Self-esteem	–0.065	0.183	0.015	0.937	0.654	1.343
Loneliness	0.141	0.033	0.001	0.869	0.814	0.927
Depression	0.193	0.106	0.005	0.825	0.670	1.015
Perceived stress	0.001	0.020	0.043	0.999	0.960	1.039
Active coping	–0.022	0.028	0.426	1.022	0.968	1.079
Passive coping	0.048	0.018	0.007	0.953	0.920	0.987

## Discussion

China has become the topmost destination for international students, but little is known about this escalating and diversified community of higher education in China. Compared with the smartphone addiction rate of different populations in various countries, the risk of smartphone addiction among international students in China is higher (Jiang et al., 2018). The present study interposes empirical evidence relating to the psychological mechanism of development and maintenance of smartphone addiction in the light of the I-PACE model. The I-PACE model includes pre-disposing variables, affective and cognitive responses to internal or external stimuli, executive and inhibitory control, and decision-making behavior, resulting in the practice of specific internet applications (Brand et al., 2016). With an increasing interest in the I-PACE model, to our knowledge, no study to date has yet assessed I-PACE-based smartphone addiction among international students of various cultural origins in a collectivistic culture or else attempted to integrate all the components of the I-PACE model in single research about smartphone addiction.

The current study illustrated that the prevalence rate of smartphone addiction in international students was 20.3%. Similarly, a Chinese study (2018) revealed that international students in China are at high risk for severe loneliness and smartphone addiction, more than 5% of the participants reported intense loneliness, and more than half of the participants exhibited smartphone addiction symptoms (Jiang et al., 2018). Moreover, we found no significant association between smartphone addiction and gender, but the number of smartphone addicts was higher among males (59%) than females (41%). Similar findings were reported by Devís-Devís et al. (2009). In contrast, some researchers have reported that female participants have more prevalence of smartphone addiction than males (Tavakolizadeh et al., 2014; Demirci et al., 2015; De-Sola Gutiérrez et al., 2016). However, some studies observed no significant difference between females and males regarding smartphone addiction (Yen et al., 2009; Dixit et al., 2010; Ahmed and Qazi, 2011). A large number of male participants in our study could explain why a high proportion of males were addicted. Furthermore, additional studies still need to unravel the inconsistent prevalence of smartphone addiction in males and females.

There was no significant association between education and smartphone addiction, but smartphone addiction varied by education. Specifically, we found that users displayed the highest smartphone addiction levels with the lowest educational attainment. Our results are consistent with the evidence suggesting that adolescents seem to be more addicted to smartphones than other age groups (Selian and Srivastava, 2004; Bianchi and Phillips, 2005; Assabawy, 2006; Wajcman et al., 2007; Attamimi, 2011; Ishii, 2011; Divan et al., 2012; Goundar, 2012; Samaha and Hawi, 2016). It’s not entirely clear why smartphone users with relatively low education levels are more addicted; Maybe these adolescents have poor self-regulation ability to compulsive use of smartphones. Our research also found that students living in dormitories were more addicted to smartphones than students living with their families. Considering previous researches on the theme, Moore and Schultz’s study (1983) yields a similar result (Moore and Schultz, 1983). The logic behind the scenes may be students who were alone and stay away from home use more smartphones to keep themselves busy and overwhelm loneliness.

Almost half of the participants in the research group stated the main reason for using smartphones was internet surfing. Our results are consistent with a previous survey conducted by Toda et al. (2004) which found that messaging and connecting to the internet were the most common causes of using smartphones (Toda et al., 2004). Furthermore, Roberts et al. (2014) also reclaimed that females use smartphones for messaging and social networks (Nazir, 2017). Similarly, Albiar and his co-workers (2012) explored that for males, smartphone use is simultaneously based on text messages, voice conversations, and surfing internet applications (Billieux et al., 2008; Albiar et al., 2012). In our study, self-esteem was negatively correlated with smartphone addiction. This finding is congruent with the cognitive-behavioral model of pathological usage of the Internet, which indicates that people with maladaptive cognitions such as low self-esteem are inclined to get addicted to the internet (Davis, 2001). Our results are braced by some empirical studies, which show that low self-esteem can predict smartphone addiction (Walsh et al., 2011; Hong et al., 2012; Park and Kim, 2015).

According to our research, smartphone addiction has a significant positive relationship with neuroticism and a significant negative correlation with psychoticism, extroversion, and social desirability (Lie personality). Likewise, a study conducted on Chinese adolescents reported that those who were addicted to the internet scored higher on the neuroticism and psychoticism dimensions of personality while scoring lower on the Lie dimension (Cao and Su, 2007). Furthermore, another survey verified that typical internet addicts exhibit anxiousness (neuroticism) and social contact problems (Van Rooij et al., 2010). Eysenck and Eysenck (1975) findings also support our study, that is, people who scored high on the Lie scale tend to score low on psychoticism and extraversion. Highly neurotic people are often afraid, sad, embarrassed, nervous, stressed, and anxious (McElroy et al., 2007; Devaraj et al., 2008), they use more smartphones to overwhelm stress and keep themselves busy. Moreover, Montag et al. (2010) found a significant negative correlation between internet addiction and the Lie dimension.

Affective components (Loneliness and Depression) of the I-PACE model have a strong positive correlation with smartphone addiction, among other components. Our verdicts resonate well with prior results from multiple studies that looked at the relationship between affective psychological traits (Depression and loneliness) and smartphone addiction. Association between smartphone addiction and depression in this study corresponds to another study among university students in Turkey by Orsal et al. (2013) who reported an alarming association between phone addiction and depression. Smartphone addiction was detected to increase as the severity of depression increased among the students (Chen and Katz, 2009; Thomée et al., 2011; Harwood et al., 2014). The link between Depression and smartphone addiction may not just be established among university students, but it may apply to the general adult population. Due to a sense of disconnection from both the host and their native countries, international students have to deal with and endure a certain level of loneliness; due to this, they become smartphone-addicted (Wei et al., 2005; Janta et al., 2014; Matar Boumosleh and Jaalouk, 2017). In a study of Chinese university students, loneliness was positively associated with depression, which emerged as the most potent independent trigger of smartphone addiction (Bian and Leung, 2015). Similarly, in a Turkish university student survey, loneliness demonstrated a significant positive association with smartphone addiction. It appeared as an independent indicator of the cyberspace-oriented relationship score (Enez Darcin et al., 2016).

In addressing the significant positive relationship between smartphone addiction and perceived stress, outcomes were consistent with the opinion of Beranuy et al. (2009) who believed that using a smartphone in a situation of stress can be looked upon as a form of substitute gratification or as a kind of addiction (Beranuy et al., 2009). The more stress students perceive, the more likely they are to become addicted to the smartphone. This is consistent with the general strain theory which holds that all kinds of strain or stress experienced by individuals will lead to negative emotions and then lead to problem behavior (Jun and Choi, 2015). Many studies have shown that stress is an important risk factor for individual addictive behavior (Mai et al., 2012; Wang et al., 2021). The influence of perceived stress on smartphone addiction has begun to gain empirical support (Chiu, 2014; Yang et al., 2020). Correspondingly, researchers also linked smartphone usage as a way of coping with stress and contentment with life (Lepp et al., 2014, 2015). So, the perceived stress has positive predictive power for smartphone addiction (Chiu, 2014).

Based on a bivariate logistic model, the possibility of having smartphone addiction symptoms was significantly higher in those international students who had a neurotic personality, low self-esteem, loneliness, depression, stress, and passive coping style. Passive coping was positively linked with smartphone addiction, while active coping was negatively related. The reason behind this may be that those who are more vulnerable to stress have passive coping strategies and use more smartphones to overcome stress (Brand, 2017). Likewise, previous surveys have shown a moderate positive association between negative coping style and smartphone addiction in students (Zhao et al., 2017) and a moderate negative association between active coping style and smartphone addiction (Li et al., 2016; Yang et al., 2017). Results were also consistent with previous findings, suggesting that emotion-focused or negative coping style is a significant predictor of internet addiction (Sugiarta and Dewi, 2021).

Overall results supported the I-PACE model that specific personal characteristics (low self-esteem, neuroticism, and social desirability), affective (loneliness and depression), cognitive (perceived stress), executive (passive coping style) components resulted in adverse emotional reactions through the perception of the situation and lead to certain addictive tendencies (Brand et al., 2016).

Using a smartphone can take people away from the social world to the world of fantasy. While the smartphone provides an excellent opportunity to socialize on the surface, they fetch people into a large virtual world. Students’ communities, whose emotional and cognitive development has not yet been completed, are heading away from the real world and constructing a virtual world for themselves. Moreover, with the extent of their absorption into the charisma of this virtual world, they move away from their families and friends and become more depressed. Also, within the virtual world of smartphones, In addition, they become depressed and expectant in the virtual world of smartphones. They express their thoughts and feelings in a few words superficially and cannot express themselves adequately. All this demonstrated that the longer students stay in the virtual world they set up for themselves, the more depressed they will be in this situation (Çağan et al., 2014).

When explaining the findings of this study, some limitations should be considered. Firstly, the research is based on participants’ self-reports, which may be vulnerable to common-method variance. Future researchers could correct this bias by measuring smartphone addiction and other dimensions behaviorally. Secondly, this study was cross-sectional and cannot infer causality. Further studies should apply longitudinal or experimental designs to confirm the causal assumptions in this study. Thirdly, the model in this study was tested on a sample of college students rather than a clinical sample. Thus, these results should not be generalized in other samples. Future research can benefit from testing the model in other samples, such as the clinical samples. Lastly, the sample was not large enough, and the sample was collected from Harbin, China, only. So, we cannot generalize it to the whole, and the questionnaires were too large, which was very difficult for respondents. Thus, future studies can expand the size of the samples and shorten the inventory size to validate the findings.

## Conclusion

Our research contributes to the literature by examining the psychological mechanisms of smartphone addiction development based on the I-PACE model. Multiple social, psychological, and cognitive factors accelerate the mechanism of smartphone addiction. In China, international students are a high-risk group for smartphone addiction. Loneliness, depression, neurotic personality, passive coping, low self-esteem, and perceived stress are menace factors for smartphone addiction. Smartphone addiction intrudes on their academic performance and affects their mental and physical health to varying degrees. It would be beneficial for the education department to guide students to use smartphones scientifically and rationally to improve their physical and mental health. It would also direct students to establish good social adaptability for happy and smooth completion of university study and life escort. This finding would have some important implications for policymakers who develop means to prevent and intervene in smartphone addiction among college students. It would be also advantageous for mental health improvement and clinical settings.

## Data Availability Statement

The raw data supporting the conclusions of this article will be made available by the authors, without undue reservation.

## Ethics Statement

The studies involving human participants were reviewed and approved by Harbin Medical University. The patients/participants provided their written informed consent to participate in this study.

## Author Contributions

AM designed the study, interpreted and analyzed the data, and drafted the manuscript. TB and WW contributed to data analysis and data interpretation. VZ, NA, SS, EZ, XQ, XY, ZQ, JZ, and YY helped draft the manuscript. All authors contributed to the design of the survey and read and approved the final manuscript.

## Conflict of Interest

The authors declare that the research was conducted in the absence of any commercial or financial relationships that could be construed as a potential conflict of interest.

## Publisher’s Note

All claims expressed in this article are solely those of the authors and do not necessarily represent those of their affiliated organizations, or those of the publisher, the editors and the reviewers. Any product that may be evaluated in this article, or claim that may be made by its manufacturer, is not guaranteed or endorsed by the publisher.
